# Supplementary material for: Contextual and individual factors associated with public dental services utilisation in Brazil: A multilevel analysis
Source: PLoS One. 2021 Jul 9;16(7):e0254310. doi: 10.1371/journal.pone.0254310 (PMC8270158; doi:10.1371/journal.pone.0254310)
Supplement: Attachment — Submitted filename: Response to reviews.docx [file pone.0254310.s001.docx]

Dear Dr Ratilal Lalloo

Thank you for allowing us to submit a revised draft of our manuscript titled “Contextual and individual factors associated with public dental services utilisation in Brazil: a multilevel analysis” to Plos One. We appreciate the time and effort that you and the reviewers have dedicated to providing your valuable feedback on our manuscript. We are grateful to the reviewers for their insightful comments on our paper. We have been able to incorporate changes to reflect most of the suggestions provided by the reviewers. We have highlighted the changes within the manuscript. We consider all the comments presented and believe that the fulfilment of the requests resulted in an improved manuscript. We hope this new version of the article is added in quality and has the opportunity to be published in this journal.

All changes are explained below in response to the reviewer's comments.

**Comments from Reviewer 1**

**Comment 1:** This sets out the history of primary care teams in Brazil and how the changing commitment to this policy has impacted patient access. However, it does not clearly set out where there is a gap in knowledge that this research paper fill. I feel that some of the history could be cut back in order to drive the narrative to understand why we need to answer the research question and how this may affect public policy. Furthermore, it would be helpful if the specific individual factors that are measured were set out in the aims, reflecting back the importance of why these factors should be examined.

**Response:** We agree with this and have incorporated your suggestion in the manuscript. We redesigned the entire introduction section taking into account the suggestions made by the reviewers.

**Comment 2:** Paragraph 1 – which middle income countries? Need to cite these or explain situation in Brazil only

**Response:** We agree with this and have incorporated your suggestion. The paragraph has been modified. *“ Brazil is a middle-income country with universal healthcare system covering dental assistance for all citizens. In 2003, the Brazilian oral health care service was transformed by the National Oral Health Policy implementation, expanding primary care teams and emphasizing the primary care-based model (1).”*

**Comment 3:** Paragraph 2 – try to avoid run- on of sentences across paragraphs. Line 4 I think should read ‘over time’

**Response:** We agree with this and have incorporated your suggestion. The paragraph has been modified.

**Comment 4:** Paragraph 3 – claim of out of pocket and private insurance plans needs citing.

**Response:** We agree with this and have incorporated a reference for this affirmation.

**Comment 5:** Paragraph 4 – first sentence reads poorly; I am not sure if this means that there is poor equity of access or good equity? The claim in the second sentence of this paragraph seems to answer the research question but does not clearly explain if this results from the findings of this survey or a previous one in the text.

**Response:** We agree with this and have incorporated your suggestion. The paragraph has been modified.

**Comment 6:** Methods – This section sets out how the dependent variable of service utilisation is related to the independent variables of age, sex, skin colour/race, education level, household income, household area, primary healthcare enrolment, type of dental attendance, self-rated oral health, number of lost teeth, HDI, average income per capita, GINI index, and oral health coverage in primary care. Omission of outliers is explained and justified. Methods of univariate and multivariate analysis are set out well.

**Response:** Thank you for these comments.

**Comment 7:** Paragraph 2 - PSU is used without first setting out meaning of acronym. It is also unclear if the 94114 home interviews relates to each member of a household – i.e. does each member of the household self-report in an interview?

**Response:** We agree with this and have incorporated your suggestion. The paragraph has been modified. The full term was included before the acronym. We further detailed the study sample in order to clarify which study participants were participating.

**Comment 8:** The interview question is set out as only ‘when did you last visit a dentist’, however the variables seem to explain much more detail such as type of dental attendance, the self rated oral health and the number of lost teeth. Is this question an umbrella for a list of further questions? I am unsure if these are asked a survey or the interview. It would be helpful to explain in detail where what questions were asked, by whom and when.

**Response:** We agree with this and have incorporated your suggestion. The paragraph has been modified. We provide all the details about the questions relevant to the definition of study participants.

**Comment 9:** Deterministic linkage technique and population expansion methods are mentioned in text but not cited or described.

**Response:** We agree with this and have incorporated your suggestion. We rewrite the text in order to detail the procedures adopted and include references about the techniques used.

**Comment 10:** Results.-Descriptive analysis paragraph 2 – it would help the reader to set out that the characteristics mentioned are the most common for each respective variable.

**Response:** We agree with this and have incorporated your suggestion. The paragraph has been modified to add this information.

**Comment 11:** The title of table 3 could do with a brief description of each model to ensure that the reader is able to quickly associate the findings with the model without referring back to the text.

**Response:** We agree with this. Nevertheless, we thought it best to include the requested information in the table caption as the title would be too long.

**Comment 12:** The tables generally read well, and the figures seem to add up to expected amounts.

**Response:** Thank you for this comment

**Comment 13:** Discussion. In the discussion, it is argued that this study shows the reorientation of dental services has been effective in increasing public dental services access, however there is no indication of what previous levels of access were like for the groups that are mentioned. Please clarify how this claim can be supported in the text.

**Response:** You have raised an important point here. Before implementing the National Oral Health Policy, public dental services were scarce for the general population. Due to this, it was not an issue addressed in health surveys, and thus there are no previous data about this topic. For this reason, when information about the use of dental services is mentioned, it is not restricted to public services.

**Comment 14:** In more of a philosophical point, which I am interested to ask the authors, how far do you think the goal of equity of access should be important? The results suggest that those with the highest need have better access to dental services, suggesting that there is some level of equity of service provision and that inequalities are mitigated. However, is the level of service provision provided good enough, or is this just equitably poor? Does this study suggest the need for increased access and increased funding, or is the current level acceptable? How might policy need to change to improve service provision futher?

**Response:** Thank you for this suggestion. It would have been interesting to explore this aspect. Considering the relevance of this comment, we have included a final paragraph that addresses these issues. We believe that this change added quality to the manuscript.

**Comments from Reviewer 2**

**Comment 1:** Abstract: Consider giving a brief overview of the study design in the abstract. It wasn’t clear when did the participants had their first dental visit vs interview? Please correct the spelling and grammar use. Please consider using a culturally sensitive term. Illiterate can be considered stigmatising or derogatory.

**Response:** Thank you for this suggestion. We modified the abstract in order to include the requested information. We consider it essential to present the study design in this section. Three independent reviewers performed a thorough language review in order to reduce grammatical errors. We review the term "illiterate" despite the term being used in previously published articles about the topic. We replaced it with the term "unlettered". We hope it is culturally appropriate.

**Comment 2:** Introduction: Please consider expanding on the reasons of lack of dental service utilization despite of available public dental services. Please consider giving some contextual information on the unmet oral healthcare needs of Brazilian population? What are the reasons? Then talk about the expansion of dental public health program and introduce your research questions/objectives. Please consider including information about about A&N model. Its main parameters and application in public health dentistry. So far, it’s completely missing and lacks clarity for your readers.

**Response**: We agree with this and have incorporated your suggestion in the manuscript. We redesigned the entire introduction section taking into account the suggestions made by the reviewers. Additionally, we added information about the Andersen Behavioral Model used in the study.

**Comment 3:** Results: Very well described while fully incorporated the A&N model as the theoretical framework.

**Response:** Thank you for this comment

**Comment 4:** Discussion: when comparing your results with several countries, what countries are you talking about? Please elaborate.

**Response:** Thank you for this suggestion. We have changed this part of the text, and we believe that the current form is better suited to the information we would like to present in the manuscript.

**Comment 5:** Your argument regarding the social gradient and utilization of dental services reads a bit misleading. Consider elaborating on the fact that utilization could be due to emergencies, symptomatic Tx, etc.

**Response:** Thank you for this suggestion. It would have been interesting to explore that aspect. However, the argument presented is plausible considering the data presented. The social gradient mentioned concerns the dose-response effect found. Concerning the need for care due to emergencies or symptoms, it is expected that it occurs among public and private services users and thus would not interfere with the results obtained.

**Comment 6:** Please review the discussion section thoroughly as there is “flaw” in the flow of ideas/arguments. Please pay close attention to word smiting and presentation of your ideas as it will be easier for the readers to follow.

**Response:** Thank you for this suggestion. We revised the entire text in order to overcome the weaknesses mentioned. We believe it resulted in a better quality manuscript.

**Comment 7:** It is not clear why are you comparing Brazil with Canada? If you are comparing a publically vs private dental care delivery system, then consider making it more generic rather than be so specific. We agree with this comment, and removed this paragraph because the idea presented was confusing.

**Comment 8:** Overall, it is an important research and will add valuable information about the oral health utilization patterns of Brazilian population.

**Response:** Thank you for this comment.

We look forward to hearing from you in due time regarding our submission and to respond to any further questions and comments you may have.

Sincerely,

Maria Helena Galvão, Arthur Medeiros, Angelo Roncalli.
